# Prevalence and Influencing Factors of Tobacco Use Among Physicians in Madinah City, Saudi Arabia, in 2024

**DOI:** 10.7759/cureus.74850

**Published:** 2024-11-30

**Authors:** Hisham Alsawadi, Abdullah M Al Saloum, Amani H Alrawi, Basant M Abdelrazek Mohamed, Kumay S Halawa, Umm e Habiba U Baqrain, Saeeda S Nawab

**Affiliations:** 1 General Medicine, Deryaq Medical Complex, Khamis Mushait, SAU; 2 Medicine, King Salman Bin Abdulaziz Medical City, Madinah, SAU; 3 Faculty of Medicine, National Ribat University, Khartoum, SDN; 4 Faculty of Medicine, International University of Africa, Khartoum, SDN; 5 Faculty of Medicine, Sulaiman Alrajhi University, Madinah, SAU; 6 Faculty of Medicine, Dow Medical University, Karachi, PAK; 7 Internal Medicine, King Salman Bin Abdulaziz Medical City, Madinah, SAU

**Keywords:** kingdom of saudi arabia (ksa), madinah city, physician tobacco use, smoking, tobacco habit, tobacco use influencing factors, tobacco use prevalence, tobacco users

## Abstract

Background

Smoking is recognized as a major public health issue globally; it is widely distributed among people of various origins and races in the world despite hard efforts on cessation programs. Its health hazards extend to dangerous complications, which mostly end in death according to statistics around the world. Tobacco use is influenced by several factors, which may include social pressures from peers, family influences, and media portrayals of smoking. This study aimed to identify the smoking prevalence among Madinah physicians, the influencing factor of their tobacco use, and their tobacco-related attitudes.

Methods

A cross-sectional study was conducted involving physicians working in selected district hospitals in Madinah. Participants were recruited using a convenience sampling technique and were asked to complete a self-administered questionnaire in English. Statistical analysis was performed using SPSS software, employing a chi-square analysis to assess smoking prevalence and influencing factors.

Results

The study involved 427 physicians, with 206 (48.2%) Saudi and 277 (64.9%) male. Most participants' ages ranged between 25 and 34 (38%). Smokers accounted for 85 (19.9%) participants, while previous smokers numbered 20 (4.7%). Male gender was significantly associated with smoking. Stress and social influence were the most common influencing factors, 62 (44.2%) and 26 (20.9%), respectively. The most common types of smoking products used among both groups were electronic cigarettes (42, 35.6%), cigarettes (40, 32.2%), and water pipes (22, 19.2%). Most physicians agreed on the importance of physicians serving as role models for the community representing 52 (61.2%) of tobacco users and 286 (83.6%) of non-tobacco users.

Conclusion

This study provides valuable insights into the prevalence, patterns, and implications of tobacco use among physicians in Madinah. The findings underscore a significant public health concern, revealing that a notable proportion of physicians are current or former tobacco users. Key demographic factors such as gender, age, and nationality demonstrate clear associations with tobacco use, highlighting the need for targeted interventions tailored to these specific groups.

## Introduction

Smoking is recognized as a major public health issue globally. According to the World Health Organization (WHO), tobacco smoking is the second leading cause of death, responsible for approximately six million deaths annually. It is the fourth most common preventable risk factor for non-communicable diseases. Various strategic and preventive measures have been implemented to address this escalating epidemic. Consequently, numerous studies have been conducted to assess and monitor smoking prevalence globally [1].

Around the world, there are 1.3 billion users of various types of tobacco, tobacco kills more than eight million people each year, seven million of those deaths are the result of direct tobacco use, and 1.3 million nonsmokers who are exposed to secondhand smoke, tobacco use contributes to poverty by diverting household spending from basic needs, around 80% of tobacco users are in the low- and middle-income countries [2].

In 2019, 19.8% of the population of Saudi Arabia are using tobacco products. Men had a significantly higher usage rate of 30.0% compared to 4.2% for women. Of these users, 17.9% smoked tobacco, with men 27.5% more likely to smoke than women 3.7%. Daily smoking was reported by 15.2% of the population, mainly among men 24.0%, and a smaller percentage of women 2.0%. Cigarette smoking was also common, with 15.9% of individuals using them. In addition, 0.8% used electronic cigarettes, and 2.4% used smokeless tobacco [3].

The prevalence of tobacco use among physicians has seen a notable decline over recent decades, particularly in high-income countries, where increased awareness of smoking's health risks and the influential role of physicians as health role models have driven this change [4]. In the United States, the smoking rate among physicians is now approximately 1-2%, significantly lower than the 14% observed in the general population [4]. In Saudi Arabia, the smoking rate among physicians is estimated at 34.1% [1]. Nevertheless, there are substantial global variations, with higher smoking rates persisting in low- and middle-income countries such as China and parts of Eastern Europe [5]. In addition, smoking rates differ across medical specialties and genders, with male physicians and certain specialties like surgeons and psychiatrists historically exhibiting higher rates of smoking [6]. Factors influencing physicians' smoking behavior include workplace stress, cultural norms, and environmental factors [7]. Efforts to further reduce tobacco use among physicians emphasize improved education, smoke-free workplace policies, tailored cessation support programs, and encouraging physicians to take on leadership roles in anti-smoking advocacy [5].

Several factors influence physicians' tobacco use. These include social pressures from peers, family influences, and media portrayals of smoking. Starting smoking at a young age, the medical specialty a physician chooses, workplace conditions, and the physician's smoking status all play crucial roles. Regional differences in smoking rates among healthcare professionals, along with various lifestyle and cultural factors, also contribute to smoking habits. In addition, there's a need to evaluate the effectiveness of smoking cessation methods tailored specifically for physicians. Understanding these influences is essential for developing targeted cessation programs, helping physicians quit smoking, and improving their ability to assist patients with smoking cessation [1].

Several studies have been conducted to estimate the prevalence of smoking among physicians, indicating that smoking is prevalent among doctors, particularly among men and individuals over 55 years old. Despite being aware of the negative consequences and future risks associated with smoking, a majority will continue to smoke [8]. In Riyadh, a study comprising 290 physicians, predominantly Saudi and male, found a smoking prevalence of 34.8%. Significant factors linked to smoking included having a smoking family member/friend, being a resident, specific medical specialties, and frequent on-call duties [1]. Waterpipe smoking is also common among Saudi physicians, especially among men and surgical specialists. The same study found that non-smokers are more likely to believe that physicians should serve as role models [9].

## Materials and methods

Study design

This cross-sectional study was conducted at the beginning of 2024 in Madinah City, Saudi Arabia, and the data were collected between January and March 2024.

Study population

All physicians and medical interns were invited in person and through the respective hospital administrations to participate in the electronic survey using a self-administering Google Forms plate (Google LLC, Mountain View, California, United States), where the randomization of the sample was preserved. The study population was invited from major governmental hospitals: King Fahad Hospital & Madinah Cardiac Center, King Salman Bin Abdulaziz Medical City, Maternity and Children's Hospital, Ohod Hospital, National Guard Hospital, Al-Amal Psychiatric Hospital, Al-Haram Hospital, Almeqat Hospital, and private hospitals (Saudi German Hospital, Al-Hayat National Hospital, Mouwasat Hospital, Madinah National Hospital, Al-Zahra Hospital, and Dr. Hamid Sulieman Al-Ahmadi Hospital).

Inclusion and exclusion criteria

All physicians working in Madinah City and medical interns were included in the study at the time of collection. By contrast, any medical students, healthcare workers rather than physicians, or physicians outside Madinah City at the time of data collection were excluded. A total of 435 responses were collected, and 427 were included in the current analyses based on the above criteria.

Quality assurance

The survey did not include physicians' data that might disclose their identity to the researchers or drive them to uphold certain information. In addition, Google Forms did not allow any missing fields of the questionnaire or more than one response to the same email, so, the chance of missing or duplicated data was eliminated. Consequently, sampling bias was prevented.

Sampling technique

A cross-sectional questionnaire-based study was conducted among physicians in Madinah City using a Google Forms electronic questionnaire (see Appendix) distributed between physicians at hospitals and through social media groups. We used a non-probability convenience sampling technique.

Sample size

The sample size of 427 was sufficient to survey a population of physicians (3,900 in the Madinah region) [10] with a 95% confidence and 5% margin of error. The required calculated sample size for the study was 350 based on the OpenEpi version 3 calculator (online open source; Open Source Epidemiologic Statistics for Public Health, www.OpenEpi.com).

Data collection

Exposure of smokers and previous smokers to influencing factors of smoking was assessed with the question: What influenced you to start using these products? Participants reported their answers with the following options: peer pressure, stress relief, social or recreational use, curiosity, family influence, advertising or media influence, and others (specify). 

The variables from all the participants were as follows: age (less than 25 years, 25-34 years, 35-44 years, 45-54 years, 55 years, and older), gender, nationality, position (intern, general practitioner, resident physician, specialist, and consultant), working sector (governmental and private), working place, current smoking (smoker, previous smoker, and non-smoker), physicians' beliefs of their role-modeling for the community, and whether they should refrain from using tobacco products to be a positive example for the community and their awareness toward tobacco use risks.

In addition, the participants who are currently smoking were asked about the type of tobacco product they are using (cigarettes, smokeless tobacco -snuff/chewing, hookah/shisha or water pipe, cigars, electronic cigarettes/vape, and others), how often they use these products (one to five times/day, six to 10 times/day, 10-20 times/day, >20 times/day, one to three times/week, three to five times/week, one to three times/month), how long they have been smoking (one to five years, six to 10 years, 10-20 years, >20 years), any previous attempts to quit, and if they currently have a quit plan.

Participants who previously smoked were asked about the type of tobacco product they used (cigarettes, smokeless tobacco - snuff/chewing, hookah/shisha or water pipe, cigars, electronic cigarettes/vape, others), how often they used these products (one to five times/day, six to 10 times/day, 10-20 times/day, >20 times/day, one to three times/week, three to five times/week, one to three times/month), how long they had been smoking (one to five years, six to 10 years, 10-20 years, >20 years), and how many attempts they made to quit smoking.

Data analysis

The statistical analysis was carried out using IBM SPSS Statistics for Windows, Version 26.0 (released 2019, IBM Corp., Armonk, NY). The variables were checked for accuracy before the data analysis was undertaken. The variables, like physicians' beliefs of their role-modeling for the community and whether they should refrain from using tobacco products to keep a positive example for the community, were recorded into agree (reference), neutral, and disagree, by collapsing “strongly agree” with “agree” and “strongly disagree” with “disagree." Similarly, awareness toward smoking-related health problems was recorded as well-informed, moderately informed, and poorly informed, by collapsing “somewhat informed” with “not informed at all." All sample characteristics were categorical and summarized using frequencies and percentages. The prevalence of smoking was compared across the strata of the prior exposure variable (i.e., influencing factors) and the relevant covariates (i.e., age, gender, nationality, working sector, working position, awareness toward smoking-related health problems, and physicians' beliefs of their role modeling for the community). The chi-square test (X^2^) was used for all the variables. Logistic univariate and multivariate regression analyses were used to model the predictors of smoking prevalence among different groups. The significance level was set at 5%.

## Results

The study sampled 427 physicians in Madinah, Saudi Arabia. The majority of physicians were males (277, 64.9%). The age distribution is mainly between 25 and 34 years (163, 38.2%). A large number of participants were non-Saudi (221, 51.8%). Most physicians were working in the governmental sector (324, 75.9%). The widest position of physicians was the consultant position (124, 29.0%) (Table 1). The comparison of the demographic characteristics between smokers and non-smokers is presented in Table 2.

**Table 1:** Demographic characteristics of a sample of physicians (n = 427) in Madinah, Saudi Arabia.

N/A		n (%)
Gender	Male	277 (64.9)
	Female	150 (35.1)
Age	Under 25 years	34 (8.0)
	25-34 years	163 (38.2)
	35-44 years	114 (26.7)
	45-54 years	84 (19.7)
	55 years and older	32 (7.5)
Nationality	Saudi	206 (48.2)
	Non-Saudi	221 (51.8)
Working sector	Governmental	324 (75.9)
	Private	103 (24.1)
Working position	Intern	64 (15.0)
	General practitioner/resident	117 (27.4)
	Specialist	122 (28.6)
	Consultant	124 (29.0)

**Table 2:** Comparison of demographic characteristics between smokers and non-smokers (n = 427).

N/A		Current smoking status		Chi-square (p-value)
		Smoker	Non-smoker	
		n (%)	n (%)	
Gender	Male	71 (25.6)	206 (74.4)	16.212 (0.000*)
	Female	14 (9.3)	136 (90.7)	
Age	Under 25 years	6 (17.6)	28 (82.4)	17.441 (0.002*)
	25-34 years	47 (28.8)	116 (71.2)	
	35-44 years	21 (18.4)	93 (81.6)	
	45-54 years	10 (11.9)	74 (88.1)	
	55 and above years	1 (3.1)	31 (96.9)	
Nationality	Saudi	62 (30.1)	144 (69.9)	25.925 (0.000*)
	Non-Saudi	23 (10.4)	198 (89.6)	
Working level	Intern	15 (23.4)	49 (76.6)	3.305 (0.347)
	GP /Resident	26 (22.2)	91 (77.8)	
	Specialist	26 (21.3)	96 (78.7)	
	Consultant	18 (14.5)	106 (85.5)	
Working sector	Governmental	76 (23.5)	248 (76.5)	10.620 (0.001*)
	Private	9 (8.7)	94 (91.3)	
Beliefs of physicians' role modeling for the community	Agree	52 (15.4)	286 (84.6)	22.004 (0.000*)
	Neutral	18 (33.3)	36 (66.7)	
	Disagree	15 (42.9)	20 (57.1)	
Beliefs of the necessity for physicians to refrain from smoking to set a positive example for the community	Agree	53 (15.8)	283 (84.2)	23.700 (0.000*)
	Neutral	16 (27.1)	43 (72.9)	
	Disagree	16 (50.0)	16 (50.0)	
Risk awareness of smoking	Well-informed	69 (20.9)	261 (79.1)	2.059 (0.357)
	Moderately informed	11 (14.3)	66 (85.7)	
	Poorly informed	5 (25.0)	15 (75.0)	
*. The Chi-square statistic is significant at the .05 level.				

**Figure 1:**
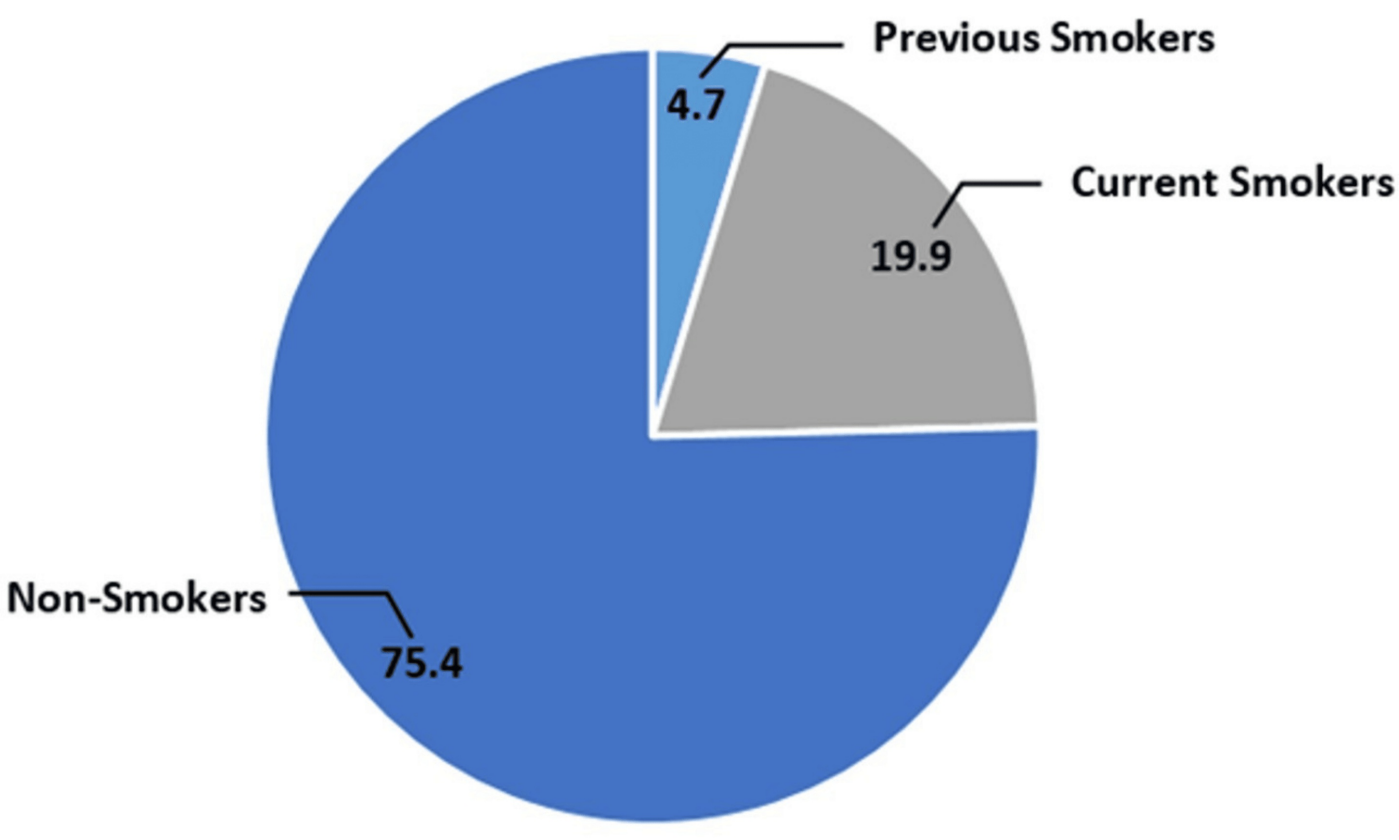
Prevalence of smoking among physicians *The statistics is significant at the 0.05 level.

**Figure 2:**
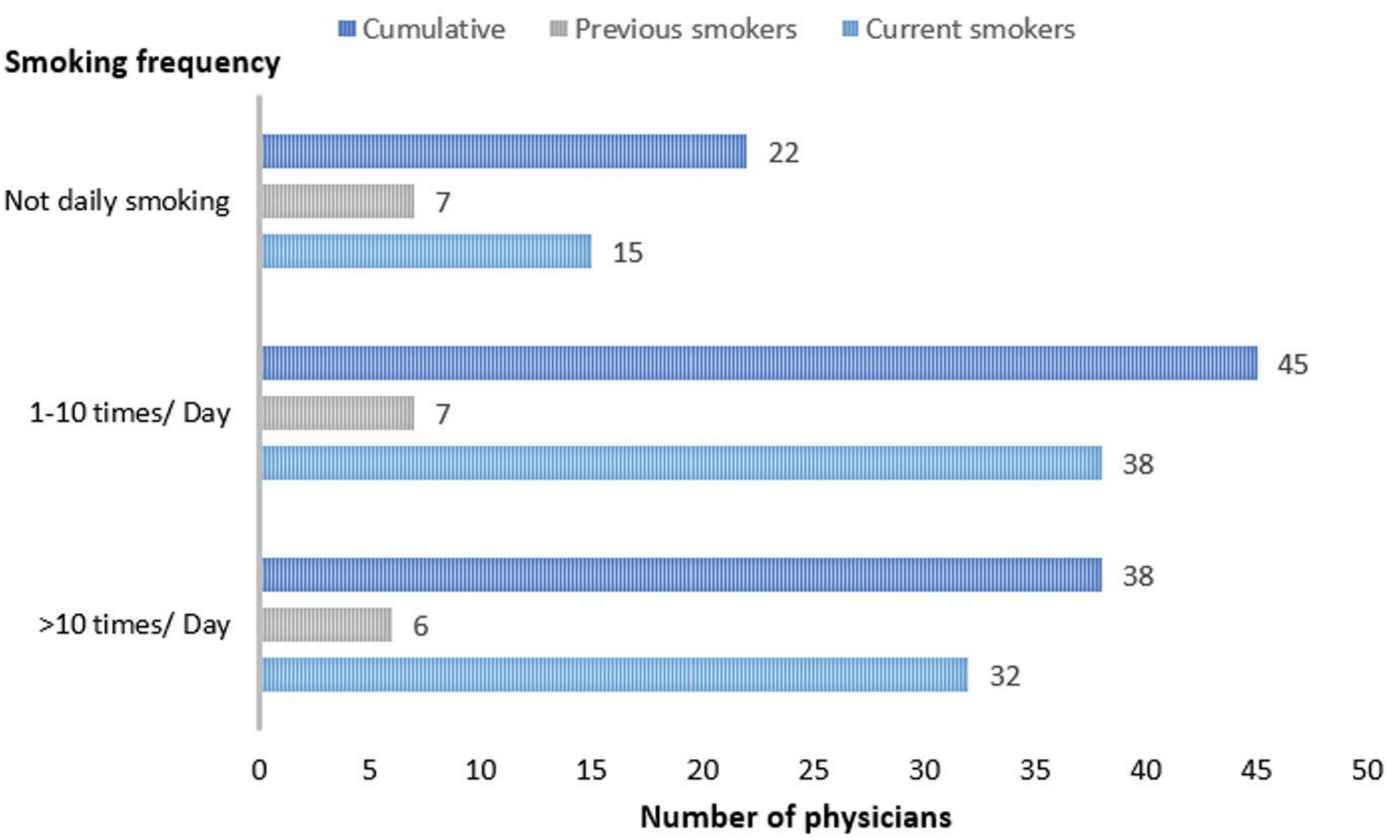
Smoking frequency among physicians *The statistics is significant at the 0.05 level.

**Figure 3:**
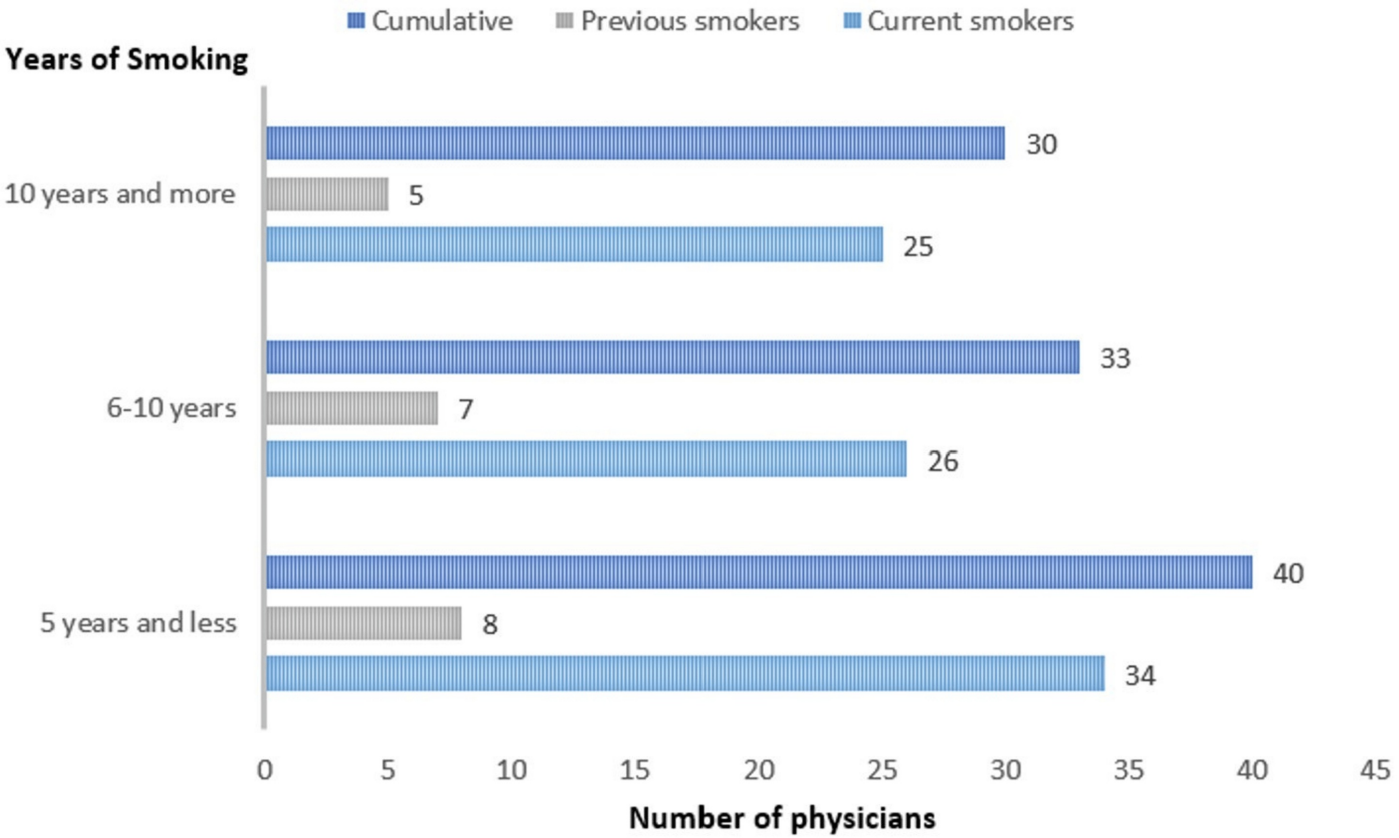
Smoking duration among physicians *The statistics is significant at the 0.05 level.

Among those physicians, current smokers were 85 (19.9%), previous smokers were 20 (4.7%), and non-smokers were 322 (75.4%). Cumulatively, smokers were 85 (19.9%), while non-smokers were 342 (80.1%) (Figure 1). The majority of smokers smoked one to 10 times per day for five years or less. In addition, 38 (44.7%) tobacco users were using tobacco up to 10 times per day while 32 (37.6%) of tobacco users were using tobacco more than 10 times per day. Moreover, 51 (60%) tobacco users have been using tobacco for six years or more. Detailed information about the frequency and duration of smoking among current and previous smokers is shown in Figures 2-3.

**Figure 4:**
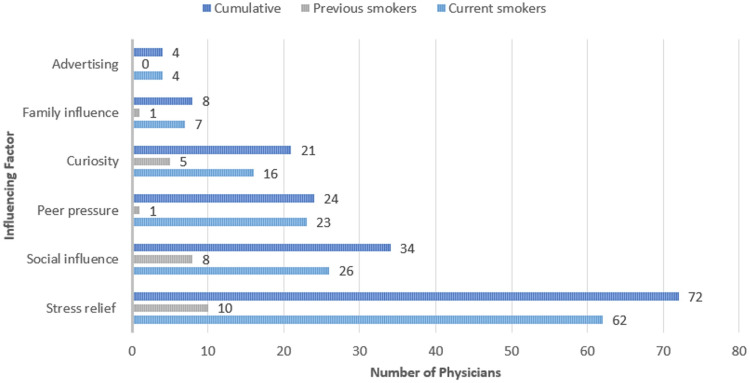
Frequency of influencing factors of smoking among physicians *The statistics is significant at the 0.05 level.

**Figure 5:**
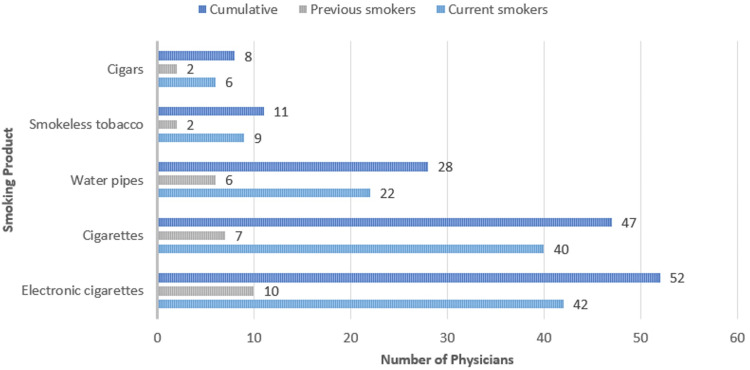
Frequency of smoking products among physicians *The statistics is significant at the 0.05 level.

The most common influencing factors among smokers and previous smokers were stress (62, 44.2%) and social influence (26, 20.9%). The most common types of smoking products used among both groups were electronic cigarettes (42, 35.6%), cigarettes (40, 32.2%), and water pipes (22, 19.2%). Detailed information about the influencing factors of smoking and the tobacco products used among current and previous smokers is shown in Figures 4-5. 

In the univariate analysis of smoking, participants' gender, age, nationality, working sector, their viewpoint of being role models for the community, and their belief in the necessity to refrain from smoking to be positive examples for the community were significantly correlated with depression (p-values < 0.05). Working level and risk awareness of smoking were not significantly correlated with smoking prevalence.

In the multivariate logistic regression of smoking, male physicians are six times more likely to be smokers than female physicians (odds ratio (OR) = 6.040; 95% confidence interval (CI): 3.006-12.135). In addition, Saudi nationality showed higher odds of 2.6 units of smoking than other nationalities (OR = 2.607; 95% CI: 1.223-5.558). Finally, physicians who disagreed or were neutral about their role modeling for the rest of the community (reference: physicians who agreed) were significantly correlated with increased odds of smoking (OR = 0.383; 95% CI: 0.155-0.951 and OR = 0.442; 95% CI: 0.205-0.954, respectively) (Table 3).

**Table 3:** Multivariate logistic regression analysis * The p-value is significant at the 0.05 level.

N/A		OR (95% CI)	p-value
Gender	Male	Reference	
	Female	6.040 (3.006-12.135)	0.000*
Age	Under 25 years	Reference	0.376
	25-34 years	0.673 (0.233-1.946)	0.465
	35-44 years	0.748 (0.235-2.381)	0.623
	45-54 years	1.144 (0.321-4.080)	0.836
	55 and above years	4.530 (0.451-45.472)	0.199
Nationality	Saudi	Reference	
	Non-Saudi	2.607 (1.223-5.558)	0.013*
Working sector	Governmental	Reference	
	Private	1.732 (0.722-4.155)	0.218
Beliefs of physicians' role modeling for the community	Agree	Reference	0.032*
	Neutral	0.442 (0.205-0.954)	0.038*
	Disagree	0.383 (0.154-0.951)	0.039*
Beliefs of the necessity for physicians to refrain from smoking to set a positive example for the community	Agree	Reference	0.060
	Neutral	0.903 (0.412-1.979)	0.799
	Disagree	0.335 (0.135-0.833)	0.019

## Discussion

Tobacco use is a serious and complex problem in today's society. Despite numerous studies on the subject, there is still a need for surveillance and investigation in areas with unknown statistics. We studied this issue to provide the community, authorities, researchers, and others with accurate data on its prevalence in this city. This information will facilitate further research and contribute to building a healthier and stronger community.

The results showed that 85 (19.9%) physicians were current tobacco users, which falls within the acceptable range compared to other studies conducted in various cities in Saudi Arabia. In addition, 20 (4.7%) physicians were previous tobacco users. In comparison, a study conducted in Abha City in 2010 reported a prevalence of current tobacco users at 18.3% and a significantly higher percentage of previous tobacco users at 15.3% [11]. A study conducted in 2015 in Makkah among PHC physicians showed that 26% were smokers and 7.6% were ex-smokers, which is higher than the findings of this study. Another study conducted in the same city and during the same year reported that 10.6% were current tobacco users and 9.8% were previous users [12,13]. A study conducted in Riyadh during 2017-2018 reported a high rate of current tobacco use among physicians, with 34.8% being current users [1]. Moreover, a study conducted in 2020 among residents in Saudi Arabia revealed that 33.5% were current smokers [14]. Unlike non-users, previous users have a higher tendency to return to tobacco use in the first two years from cessation [15], especially since many of the tobacco users in this study had been regular users for a long time and in high amounts.

The gender distribution of tobacco users demonstrated statistical significance (p-value = 0.000), with a higher proportion of male physicians (277, 64.9%) to female physicians. Among the male physicians, 71 (25.6%) were tobacco users, whereas only 14 (9.3%) female physicians used tobacco products. Another study among healthcare workers also showed statistically significant results, with 62.3% of participants being male; 27.6% of them being tobacco users and 7.2% being female tobacco users [13]. In addition, two more studies indicated that males represented most of the population, accounting for 68.7% and 60.3%, respectively. In these studies, 26.7% and 70.4% of the male participants were tobacco users, respectively [1,12]. Thus, these findings suggest a strong association between gender and tobacco use, with significantly higher smoking rates among male physicians, which may be influenced by sociocultural factors, traditional gender roles, social norms, and different stress-related coping mechanisms. The higher prevalence of smoking among men could also be due to greater social acceptance and exposure to smoking environments. These insights highlight the need for gender-specific interventions and policies to effectively address smoking among healthcare professionals.

In addition, the distribution of non-Saudi was 221 (51.8%), followed by 206 (48.2%) Saudi. As exemplified, a study among physicians in Riyadh showed that 91% of the participants were Saudi nationals, whereas the rest were non-Saudi nationals (9%), which may explain the variety of nationality distribution between Madinah and Riyadh cities [16]. Furthermore, the majority of tobacco users were of Saudi nationality (30.1%), and 10.4% were other nationalities, which was statistically significant (p-value = 0.000). Additional studies may participate in clarifying factors of widespread tobacco use among Saudi physicians. Similarly, A study presented the percentage of Saudi tobacco users as 30.5% out of 45.6% and 11.1% tobacco users of non-Saudi out of 54.4% [13]. Notably, the relationship between nationality and tobacco use among physicians shows a higher prevalence of smoking among Saudi nationals compared to non-Saudis. This trend is consistent across different studies and highlights the influence of cultural, economic, and social factors on smoking behaviors. Understanding these nuances can help the improvement of targeted public health interventions to reduce tobacco use among physicians.

The results revealed a significant prevalence of electronic cigarettes (42, 35.6%) among tobacco users, highlighting the need for further investigation to formulate effective recommendations. In addition, traditional cigarettes were also widely used, accounting for 40 (32.2%) users, suggesting common characteristics with electronic cigarettes in terms of ease of use and accessibility. In contrast, water pipes were less prevalent, representing only 22 (19.2%) of tobacco use. A study conducted among residents in Saudi Arabia in 2020 showed a higher prevalence of regular cigarettes (47.7%) compared to electronic cigarettes (30.1%), underscoring the recent popularity of electronic cigarettes. Water pipes exhibited an even lower prevalence at 11.1%, indicating a declining trend in usage [14]. By contrast, a study among healthcare workers indicated that 20.5% used waterpipes (Shisha) [11]. In addition, among physicians, 20% out of the total 34.1% were smoking waterpipes (Shisha), with the remaining 23% smoking cigarettes, as reported in another study [1]. Thus, the prevalence of different types of tobacco products among physicians highlights significant trends and shifts in smoking behavior. Electronic cigarettes lead in popularity, while traditional cigarettes remain significant due to habitual use and accessibility. Water pipes are less prevalent, likely due to their complexity and changing social habits. Understanding these patterns is crucial for developing targeted interventions to reduce tobacco use among physicians and promote healthier alternatives.

Moreover, most of these users (38, 44.7%) reported using tobacco daily up to 10 times per day, while 32 (37.6%) used tobacco more than 10 times per day. Notably, 51 (60%) had been using tobacco for six years or more. In addition, a study showed that 78.4% of tobacco users smoked every day for six months or longer, while 72.7% regularly smoked between the ages of 18 and 28 years, and 13.6% started smoking regularly before the age of 18 [12]. In addition, another study showed that the age at which 736 healthcare workers began smoking ranged from six to 25 years, with an average starting age of 18.2 ± 5.7 years [11]. In general, the high frequency and long duration of tobacco use among physicians emphasize the persistent nature of smoking habits and the significant challenges associated with cessation. The data underscores the importance of early prevention efforts and targeted interventions to reduce smoking initiation and promote cessation, particularly among younger populations. Addressing these issues is vital to mitigating the long-term health risks associated with tobacco use and improving overall public health outcomes.

Stressful conditions in the medical environment are common. However, it is surprising that such conditions can influence physicians to engage in this harmful habit, despite their awareness of the associated health hazards. Our study revealed that 62 (44.9%) of the respondents indicated that stress was the primary factor influencing their tobacco use. Social influences were the next most significant factor, impacting 26 (18.8%) of the respondents, followed by peer pressure at 23 (16.7%). Other factors included curiosity (16, 11.6%), family influences (seven, 5.1%), and advertising (four, 2.9%). Among previous tobacco users, stress remained the predominant influence at 10 (40.0%), followed by social influences at eight (32.0%), curiosity at five (20.0%), and both family influences and peer pressure at one (4.0%) each. Nowadays, electronic cigarettes receive significant media attention, similar to how traditional cigarettes were previously highlighted. This underscores the influential role of media coverage in shaping community perceptions, regardless of their level of education [17,18]. In a recent study conducted in Riyadh, researchers explored the relationship between novelty-seeking behavior and tobacco use. The study revealed that 44.5% of tobacco users attributed their smoking habits to the desire for new and fashionable experiences [16]. These findings underscore the critical issue of stress-induced tobacco use among physicians, driven by the intense pressures of their work environment despite their awareness of health risks. The significant roles of social influences and peer pressure highlight the need for a non-smoking culture in healthcare settings. In addition, curiosity, family influences, and advertising still contribute to tobacco use, emphasizing the importance of early education, preventive measures, and comprehensive public health campaigns targeting individuals, families, and communities. This recent study [16], aligned the desire for new and fashionable experiences, especially through electronic cigarettes, further underscores the need for stringent advertising regulations to curb their appeal and mitigate this growing trend.

According to the American Lung Association, the negative effects of tobacco use can extend to users' families and the community through second-hand smoke and the influence of their role modeling [19]. Regarding this, most users and non-user physicians agreed on the importance of physicians serving as role models for the community, showing a statistically significant relationship. This underscores the importance of these studies and investigations. In addition, the sampled physicians believed that physicians must refrain from smoking to set a positive example for the community, which was also statistically significant. Similarly, a cross-sectional study revealed that non-smoker doctors were more likely than smoker doctors to believe that physicians should serve as role models 79% versus 60% in Riyadh City, which shares a similar community, race, and culture with Madinah City [9]. Simultaneously, the majority of physicians were well-informed and aware of the risks and health hazards associated with tobacco use, as expected within medical communities. In addition, a study concluded that most physicians demonstrated knowledge about smoking-related topics, including the health hazards of tobacco use [12]. Therefore, the role of physicians as non-smoking role models is crucial for influencing community attitudes toward tobacco use and promoting public health. The significant agreement among both smoking and non-smoking physicians on the importance of this role underscores the need for continued efforts to support smoking cessation among healthcare professionals. By leveraging their knowledge and influence, physicians can lead by example, reducing the prevalence of tobacco use and its associated health risks within the broader community.

Implication

The study emphasizes the pivotal role of healthcare professionals as role models in tobacco cessation efforts. Addressing tobacco use within healthcare settings not only benefits the health of physicians themselves but also enhances their ability to advocate for tobacco control and promote healthier behaviors among their patients and the broader community. Recommendations stemming from this study include enhancing tobacco cessation programs specifically tailored to healthcare professionals, implementing stringent tobacco-free policies in healthcare facilities, and integrating smoking cessation counseling into routine medical practice. In addition, continued surveillance and research are essential to monitor trends, evaluate the effectiveness of interventions, and inform evidence-based policies aimed at reducing tobacco use. By addressing these recommendations, stakeholders can collaborate to create a healthier environment within healthcare settings and contribute to broader public health efforts aimed at combating tobacco-related diseases and promoting overall well-being in Madinah and beyond.

Study strengths

The study provided a wide comparison limit due to similar previous studies in other cities in Saudi Arabia despite limited previous studies in Madinah City. In addition to that, a study was conducted in a relatively short time and closed with fewer expenses. Interestingly, the study suggested valuable factors to study in the future.

Limitations

Tobacco use among physicians in Madinah City presents significant findings and implications, yet several limitations should be considered. The study's reliance on self-reported data for tobacco use may introduce biases such as social desirability or inaccurate recall. Objective measures like biochemical validation could enhance the accuracy of reported smoking prevalence. Moreover, the cross-sectional design limits the ability to establish causal relationships between influencing factors and tobacco use behaviors. Future research should consider longitudinal approaches to track changes over time and assess the effectiveness of interventions. In addition, the study's sample size and location specificity may limit the generalizability of findings beyond this particular cohort. Conducting larger, more diverse studies across different regions would provide a more comprehensive understanding of tobacco use patterns among physicians in Saudi Arabia. Addressing these limitations will be crucial in informing effective public health strategies and interventions aimed at reducing tobacco use and promoting healthier lifestyles among healthcare professionals.

Recommendations

Researchers and academic institutions should conduct thorough studies on tobacco use trends among healthcare professionals in Madinah, employing longitudinal approaches and collaborating with local healthcare providers. Healthcare institutions and administrators should enforce strict tobacco-free policies, provide cessation support for staff, and invest in tobacco control programs. In addition, government and public health authorities need to advocate for national tobacco control policies, allocate resources for public health campaigns, and integrate smoking cessation into healthcare systems. Moreover, professional associations and societies should develop guidelines for cessation interventions, raise awareness among members, and support policy changes for tobacco-free environments. Also, participation in cessation programs, advocacy for tobacco-free policies, and staying informed about tobacco control strategies by healthcare professionals are recommended. Furthermore, community organizations and advocacy groups should collaborate with healthcare providers, educate the public on tobacco risks, and advocate for comprehensive tobacco control policies. Given this, future research may distribute the prevalence among all healthcare workers and also study tobacco users' beliefs and behaviors toward electronic cigarettes. In addition, future research may discover more about the stressful conditions affecting doctors in their work environments and possible ways for stress relief appropriate for their work and environment.

## Conclusions

This study provides valuable insights into the prevalence, patterns, and implications of tobacco use among physicians in Madinah. The findings underscore a significant public health concern, revealing that a notable proportion of physicians are current or former tobacco users. Key demographic factors such as gender, age, and nationality demonstrate clear associations with tobacco use, highlighting the need for targeted interventions tailored to these specific groups.

## Appendices

Research Questionnaire 

This questionnaire assesses the prevalence and factors influencing tobacco use among physicians in Madinah, Saudi Arabia. Note: This questionnaire is specific for physicians (Consultants, Specialists, residents, interns). Note: if you are not a physician do not fill this questionnaire. Note: Please participate and fill out the questionnaire only once. Welcome and thank you for your participation. This questionnaire takes a maximum of 1-3 minutes. Please fill out the questionnaire with credibility and transparency, knowing that we will not address personal names or any private information unrelated to the research topic. Filling out the questionnaire means giving us your personal consent to collect and use the information for scientific research purposes only. This study is designed for scientific research purposes only.

Section 1: Personal Information

This section aims to collect your general information like gender, age, job title, etc.

What is your gender?

         1- Male

         2- Female

Please select your age group:

          1- (Under 25)

          2- (25-34)

          3- (35-44)

          4- (45-54)

          5- (55 or older)

What is your current occupation in the field of healthcare?

          1- Intern

          2- General Practitioner

          3- Resident

          4- Specialist

          5- Consultant

          6- Other (please specify)

Which hospital are you working in? 

          1- Madinah General Hospital

          2- Maternity and Children's Hospital

          3- Al Amal Psychiatric Hospital

          4- King Fahad Hospital of Madinah

          5- Ohod Hospital

          6- National Guard Hospital of Madinah

          7- Almeqat Hospital

          8- Al Haram Hospital

          9- King Faisal Specialist Hospital & Research Center

          10- Saudi German Hospital of Madinah

          11- Mouwasat Hospital of Madinah

          12- Dr Hamid Sulieman Al Ahmadi Hospital

          13- Madina National Hospital

          14- Hayat National Hospital.

          15- Al-Zahra Hospital

          16- Other (please specify)

Which sector are you working in?

          1- Governmental   

          2- Private

What is your nationality?

          1- Saudi

          2- Non-Saudi (please specify)

Section 2: Tobacco Use Habits

Do you use any tobacco or nicotine products?

          1- Yes ^(complete this section)^

          2- No ^(move to section 4)^

          3- Yes, but I've quit ^(move to section 3)^

If you use tobacco products, please specify which ones: ^(you can choose more than one option)^

          1- Cigarettes

          2- Smokeless tobacco (snuff, chewing tobacco)

          3- Shisha or water pipe

          4- Cigars

          5- Electronic cigarettes (e-cigarettes or vape)

          6- Other (please specify)

If you currently use tobacco products, please specify how often:

          1- (1-5) times per day

          2- (6-10) times per day

          3- (10-20) times per day

          4- More than ( 20 ) times per day

          5- (1-3) times per week

          6- (3-5) times per week

          7- (1-3) times per month

How long have you been using tobacco or nicotine products?

          1- Less than one year

          2- (1-5) years

          3- (6-10) years

          4- (11-20) years

          5- More than (20) years

What influenced you to start using these products? ^(you can choose more than one option)^

          1- Peer pressure or influence

          2- Stress relief

          3- Social or recreational use

          4- Curiosity

          5- Family influence

          6- Advertising or media influence

          7- Other (please specify)

Have you ever attempted to quit or reduce your tobacco or nicotine product use?

          1- Yes

          2- No

Do you have a plan to quit using tobacco?

          1- Yes

          2- No

*Section 3: Previous Tobacco Users* ^(skip for current tobacco users and non-tobacco users)^

If you used tobacco products, please specify which ones: ^(you can choose more than one option)^

          1- Cigarettes

          2- Smokeless tobacco (snuff, chewing tobacco)

          3- Hookah or water pipe

          4- Cigars

          5- Electronic cigarettes (e-cigarettes or vape)

          6- Other (please specify)

If you used tobacco products, please specify how often:

          1- (1-5) times per day

          2- (6-10) times per day

          3- (10-20) times per day

          4- More than (20) times per day

          5- (1-3) times per week

          6- (3-5) times per week

          7- (1-3) times per month

How long did you use tobacco or nicotine products?

          1- Less than one year

          2- (1-5) years

          3- (6-10) years

          4- (11-20) years

          5- More than (20) years

What influenced you to start using these products? ^(you can choose more than one option)^

          1- Peer pressure or influence

          2- Stress relief

          3- Social or recreational use

          4- Curiosity

          5- Family influence

          6- Advertising or media influence

          7- Other (please specify)

How many attempts did you make to quit tobacco or nicotine product use before you quit?

          1- Once 

          2- Twice

          3- (3) times

          4- More than three times

Section 4: Attitudes and Beliefs

Are doctors role models for the rest of the community?

          1- Strongly agree

          2- Agree

          3- Neutral

          4- Disagree

          5- Strongly disagree

In your opinion, should physicians refrain from using tobacco or nicotine products to set a positive example for their patients and the community?

          1- Strongly agree

          2- Agree

          3- Neutral

          4- Disagree

          5- Strongly disagree

How well-informed are you about the health risks associated with tobacco and nicotine product use?

          1- Very well-informed

          2- Moderately informed

          3- Somewhat informed

          4- Not well-informed

          5- Not informed at all

Thank you for your participation.
